# Prevalence, risk factors and associated adverse pregnancy outcomes of anaemia in Chinese pregnant women: a multicentre retrospective study

**DOI:** 10.1186/s12884-018-1739-8

**Published:** 2018-04-23

**Authors:** Li Lin, Yumei Wei, Weiwei Zhu, Chen Wang, Rina Su, Hui Feng, Huixia Yang, on behalf of the Gestational diabetes mellitus Prevalence Survey (GPS) study Group

**Affiliations:** 1 Department of Obstetrics and Gynaecology, Peking University First Hospital, Xi’anmen Street No.1, Xicheng District, Beijing, 100034 China; 2 National Institute of Hospital Administration, Beijing, 100191 China

**Keywords:** Anaemia, Pregnant women, Associated factors, Pregnancy outcomes, China

## Abstract

**Background:**

Anaemia in pregnant women is a public health problem, especially in developing countries. The aim of this study was to assess the prevalence and related risk factors of anaemia during pregnancy in a large multicentre retrospective study (*n* = 44,002) and to determine the adverse pregnancy outcomes in women with or without anaemia.

**Methods:**

The study is a secondary data analysis of a retrospective study named “Gestational diabetes mellitus Prevalence Survey (GPS) study in China”. Structured questionnaires were used to collect socio-demographic characteristics, haemoglobin levels and pregnancy outcomes from all the participants. Anaemia in pregnancy is defined as haemoglobin < 110 g/L. We used SPSS software to assess the predictors of anaemia and associated adverse pregnancy outcomes.

**Results:**

The overall prevalence of anaemia was 23.5%. Maternal anaemia was significantly associated with maternal age ≥ 35 years (AOR = 1.386), family per capita monthly income< 1000 CNY (AOR = 1.671), rural residence (AOR = 1.308) and pre-pregnancy BMI < 18.5 kg/m^2^ (AOR = 1.237). Adverse pregnancy outcomes, including GDM, polyhydramnios, preterm birth, low birth weight (< 2500 g), neonatal complications and NICU admission, increased significantly (*P* < 0.001) in those with anaemia than those without.

**Conclusions:**

The results indicated that anaemia continues to be a severe health problem among pregnant women in China. Anaemia is associated with adverse pregnancy outcomes. Pregnant women should receive routine antenatal care and be given selective iron supplementation when appropriate.

**Electronic supplementary material:**

The online version of this article (10.1186/s12884-018-1739-8) contains supplementary material, which is available to authorized users.

## Background

Anaemia is defined as a condition in which haemoglobin (Hb) level in the body is lower than normal, which results in a decreased oxygen-carrying capacity of red blood cells to tissues [1]. It affects all age groups, but pregnant women and children are more vulnerable. Stevens et al. [2] reported that the global prevalence of anaemia in non-pregnant women, pregnant women and children is 29, 38 and 43%, respectively.

According to the WHO guidelines, anaemia in pregnancy is defined as a haemoglobin level < 110 g/L [3, 4]. The prevalence of anaemia is an important health indicator. A study in 2013 showed that anaemia is more prevalent in developing countries (43%) than developed countries (9%) [5]. Previous studies have reported that the prevalence of anaemia in pregnancy varies in women with different socio-economic conditions, lifestyles, or health-seeking behaviours across different cultures [6, 7].

Anaemia is one of the most prevalent complications during pregnancy. It is commonly considered a risk factor for poor pregnancy outcomes and can result in complications that threaten the life of both mother and foetus, such as preterm birth [8], low birth weight [9], foetal impairment, and maternal and foetal deaths [10].

Physiologically, plasma volume expands by 25–80% of pre-pregnancy volumes between the second trimester and the middle of the third trimester of pregnancy [11, 12]. This induces a modest decrease in Hb levels during pregnancy. Previous studies show that the best time to investigate any risk factors associated with anaemia may be up until 20 weeks of gestation [13]. Thus, in this study, we took the haemoglobin level estimated before the 14th week of gestation to determine factors associated with anaemia in pregnant women. Considering the physiological changes in plasma volume, we used the third trimester’s Hb level to assess the pregnancy outcomes of anaemia.

In the present study, associated factors, including socio-demographic factors, body mass index, parity and age were analysed, and we evaluated the maternal and foetal outcomes among anaemic and non-anaemic women.

## Methods

### Data sources

We conducted a large retrospective study entitled “Gestational diabetes mellitus Prevalence Survey in China (the GPS study)” in 21 hospitals, including 15 centres in Beijing, 5 centres in Guangzhou and 1 centre in Chengdu. Medical records of 44,002 pregnant women who delivered between June 2013 and May 2015 were collected. We designed a structured questionnaire to collect the socio-demographic, obstetric and medical history of pregnant women [14]. An additional file shows the questionnaire in more details (see Additional file 1). The GPS study aimed to investigate the prevalence of pregnancy diseases and the factors associated with the determined diseases.

### Study design and population

The present analysis was based on data from the GPS study. We recorded the haemoglobin level of pregnant women in three trimesters. Excluding 599 cases that lacked haemoglobin values in either trimester, 43,403 pregnancies were included in the study. We demonstrated the current status of anaemia during pregnancy in China from three aspects, including the prevalence of anaemia, related risk factors and the relationship between anaemia and pregnancy outcomes.

#### Prevalence of anaemia

Since three trimesters’ haemoglobin levels of pregnant women were recorded, we found that the diagnosis of anaemia should be made when the haemoglobin value in any trimester was lower than 110 g/L. Thus, 43,403 participants were included.

#### Related risk factors

To analyse the factors associated with anaemia, we selected the Hb value of the early trimesters as a subgroup, which included 26,924 pregnant women. Women with pre-pregnancy diabetes mellitus (PGDM), chronic hypertension and chronic renal disease were excluded.

#### Anaemia and adverse pregnancy outcomes

To evaluate the risk of adverse pregnancy outcomes in women with and without anaemia, we used the Hb value in the 3rd trimester, which included 41,569 women. Women with PGDM, twin or multiple pregnancies, chronic hypertension, or other foetal factors (foetal malformations, foetal death) were excluded. We analysed the maternal outcomes, including caesarean section, GDM, hypertension, premature rupture of membranes (PROM), foetal distress, placenta abruption, polyhydramnios, and oligohydramnios; and infant complications, such as preterm labour, low birth weight, neonatal complications and NICU admission.

### Definitions

Based on WHO criteria, we defined anaemia in pregnancy as Hb < 110 g/L. Mild, moderate and severe anaemia were defined as Hb measurements between 100 and 109 g/L, 70-79 g/L and less than 70 g/L, respectively [3].

Gestational age was based on the number of days between the first day of an expectant mother’s last menstrual period (LMP) and date of delivery and was expressed in the week after the LMP. The 1st, 2nd and 3rd trimester were defined as a gestational age less than 14 weeks, 14–27.9 weeks and 28–42 weeks, respectively. Body mass index (BMI) was divided into four groups based on WHO recommendations for Asian populations: underweight: BMI < 18.5 kg/m^2^, normal: 18.5-23.9 kg/m^2^, overweight: 24-27.9 kg/m^2^, and obesity: ≥28 kg/m^2^ [15].

Definition of maternal and neonatal outcomes: macrosomia was defined as new-born birth weight ≥ 4000 g, while new-born birth weight < 2500 g represented low birth weight. Preterm birth is defined as the time of delivery between the 28 and 36^+ 6^ gestational weeks. GDM was diagnosed according to the Chinese MOH 2011 criteria [16], which recommend that the diagnosis should be made when any one of the following values is met or exceeded in the 75 g oral glucose tolerance test (75 g OGTT) at 24-28 weeks: 0 h (fasting), 5.1 mmol/L; 1 h, 10.0 mmol/L; and 2 h, 8.5 mmol/L. Hypertensive disorders include preeclampsia, eclampsia, pregnancy-induced hypertension and haemolysis, elevated liver enzymes and low platelet syndrome (HELLP).

### Statistical analysis

Data were entered into EPI data version 3.1 and cleaned. Finally, data were analysed using SPSS software version 22.0 for Mac (Chicago, IL, USA). Data were summarized in tables and figures. Continuous variables were presented as the mean ± standard deviations (SDs). Bivariate and multivariate logistic regression analyses were performed to adjust for potential confounding factors. Variables with *P*-value≤0.25 by the bivariate analysis were candidates for the multiple logistic regression model. The results of group comparisons of risk factors and pregnancy outcomes were expressed as ORs (95% CIs) for categorical variables. The *P*-value was set at < 0.05 for statistical significance.

## Results

### Prevalence of anaemia among pregnant women

**Fig. 1:**
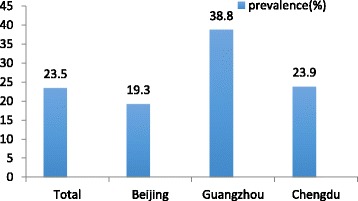
Prevalence of anaemia in China

We included 43,403 pregnant women in our study. The number of participants in the anaemia and non-anaemia groups was 10,199 and 33,204, respectively. The prevalence of anaemia in total was 23.5% (10,199/43,043). The maternal demographic characteristics are shown in Table 1. Sorted by city, we found that the prevalence of anaemia in Beijing, Guangzhou and Chengdu was 19.3%, 38.8% and 23.9%, respectively (Fig. 1).

**Table 1:** Maternal demographic characteristics. Data are expressed as the means ± SDs or *n* (%)

Variables	Anaemic	Non-anaemic	Total
Pregnant women	10,199 (23.5%)	33,204 (76.5%)	43,403 (100.0%)
Maternal age (years)	29.50 ± 4.46	29.80 ± 4.19	29.73 ± 4.25
Gravidity	1.94 ± 1.20	1.85 ± 1.13	1.87 ± 1.15
Parity	0.78 ± 0.73	0.59 ± 0.67	0.64 ± 0.69
Pre-pregnancy BMI (kg/m^2^)	20.57 ± 4.39	21.31 ± 4.41	21.13 ± 4.42
Pre-pregnancy height (cm)	54.00 ± 8.38	56.42 ± 9.18	55.85 ± 9.06
Pre-pregnancy weight (kg)	160.48 ± 4.96	161.56 ± 4.92	161.30 ± 4.95

In this study, we collected the Hb value of three trimesters, and there were 26,924, 33,879 and 41,569 effective Hb values in the 1st, 2nd and 3rd trimester, respectively. We found that the mean Hb values in the three trimesters were 129.89 ± 9.90 g/L, 118.99 ± 9.78 g/L and 121.21 ± 12.62 g/L, respectively (Table 2). The prevalence of anaemia was higher in the 2nd trimester (14.7%) and 3rd trimester (16.6%) than in the 1st trimester (2.7%) (Table 3). The severity of anaemia in pregnancy is shown in Table 3. Few pregnant women suffered from severe anaemia, while most of the participants had mild and moderate anaemia.

**Table 2:** Hb level of three trimesters in pregnancy

	Number	Percent	Hb (g/L)	Testing time (week)
1st trimester	26,924	61.2	129.89 ± 9.90	10.65 ± 2.72
2nd trimester	33,879	77.0	118.99 ± 9.78	23.17 ± 3.39
3rd trimester	41,569	94.5	121.21 ± 12.62	37.24 ± 2.71

**Table 3:** Anaemia and its severity in three trimesters in pregnancy

	Total	Anaemia		Severity of anaemia					
				Mild		Moderate		Severe	
	*N*	*N*	%	*N*	%	*N*	%	*N*	%
1st trimester	26,924	732	2.7	567	77.5	165	22.5	0	–
2nd trimester	33,879	4996	14.7	4144	82.9	848	17.0	4	0.1
3rd trimester	41,569	6888	16.6	5073	73.6	1800	26.1	15	0.2

### Factors associated with anaemia

A total of 26,255 women were included to evaluate the associated risk factors. Table 4 shows the maternal characteristics of the anaemia group versus the non-anaemia group. We found that the differences in maternal age, educational status, city, family monthly income, residence, pre-pregnancy BMI and parity were significant between anaemic and non-anaemic women (Table 5).

**Table 4:** Maternal characteristics of the selected population. Data expressed as the means ± SDs or *n* (%)

Variables	Anaemic	Non-anaemic	Total
Haemoglobin (g/L)	103.31 ± 5.88	130.55 ± 8.89	129.80 ± 9.88
Gestation week (weeks)	11.32 ± 2.38	10.63 ± 2.73	10.65 ± 2.72
Maternal age (years)	29.91 ± 4.43	30.02 ± 3.93	30.01 ± 3.94
Gravidity	1.96 ± 1.21	1.77 ± 1.09	1.78 ± 1.10
Parity	0.83 ± 0.72	0.51 ± 0.63	0.52 ± 0.63
Pre-pregnancy BMI (kg/m^2^)	20.34 ± 3.88	21.23 ± 4.13	21.20 ± 4.13
Pre-pregnancy height (cm)	160.45 ± 4.67	161.77 ± 4.91	161.74 ± 4.91
Pre-pregnancy weight (kg)	53.00 ± 7.64	56.13 ± 8.76	56.05 ± 8.75

**Table 5:** Clinical variables in association with anaemia among pregnant women [*N* (%)]

Variables	Anaemic	Non-anaemic	*χ* ^*2*^	*P*-value
Pregnant women	719 (23.5%)	25,536 (76.5%)		
Maternal age				
< 20y	5 (8.2%)	56 (91.8%)	15.424	< 0.001*
20-35	616 (2.6%)	22,850 (97.4%)		
≥ 35	98 (3.6%)	2630 (96.4%)		
Educational status				
College and above	485 (2.3%)	20,168 (97.7%)	69.346	< 0.001*
Junior or senior	224 (4.5)	4748 (95.5%)		
Primary or illiteracy	3 (3.3%)	88 (96.7%)		
Cities				
Beijing	296 (1.6%)	17,951 (98.4%)	477.503	< 0.001*
Guangzhou	164 (10.5%)	1400 (89.5%)		
Chengdu	259 (4.0%)	6185 (96.0%)		
Family per capita monthly income (CNY)				
< 1000	140 (2.2%)	6170 (97.8%)	9.256	0.010*
1000-4999	262 (2.9%)	8667 (97.1%)		
≥ 5000	308 (2.9%)	10,141 (97.1%)		
Residence				
Urban	487 (2.4%)	19,671 (97.6%)	36.801	< 0.001*
Rural	226 (3.9%)	5580 (96.1%)		
Pre-pregnancy BMI (kg/m^2^)				
< 18.5	152 (3.6%)	4046 (96.4%)	35.163	< 0.001*
18.5-23.99	483 (2.8%)	17,737 (97.2%)		
24-27.99	62 (1.7%)	3589 (98.3%)		
≥ 28	13 (1.3%)	980 (98.7%)		
Parity				
0	246 (1.7%)	14,316 (98.3%)	148.303	< 0.001*
1-3	463 (4.0%)	11,101 (96.0%)		
≥ 4	8 (11.0%)	65 (89.0%)		

The results are reported as the frequency (percentage) and *P-value< 0.05 was statistically significant

A binary logistic regression model was performed to identify the factors affecting maternal anaemia. After adjusted by other variables, maternal age ≥ 35 years (AOR = 1.386, 95%CI:1.103-1.742), women from Guangzhou (AOR = 7.293, 95%CI:5.455-9.751) or Chengdu (AOR = 2.147, 95%CI:2.174-3.777), family per capita monthly income < 1000 CNY (AOR = 1.671, 95%CI:1.291-2.162), rural residence (AOR = 1.308, 95%CI:1.095-1.563) and pre-pregnancy BMI < 18.5 kg/m^2^ (AOR = 1.237, 95%CI:1.021-1.498) were the predictors of anaemia among the pregnant women (Table 6).

**Table 6:** Predictors of anaemia among pregnant women

Variables	COR	95%CI	*P*-value	AOR	95%CI	*P*-value
Maternal age						
< 20y	3.312	1.322-8.297	0.011*	2.489	0.958-6.467	0.061
20-35	1			1		
≥ 35	1.382	1.113-1.717	0.003*	1.386	1.103-1.742	0.005*
Educational status						
College and above	1			–		
Junior or senior	0.705	0.222-2.237	0.553	–		
Primary or illiteracy	1.384	0.434-4.408	0.583	–		
Cities						
Beijing	1			1		
Guangzhou	7.104	5.826-8.663	< 0.001*	7.293	5.455-9.751	< 0.001*
Chengdu	2.540	2.144-3.008	< 0.001*	2.847	2.147-3.777	< 0.001*
Family per capita monthly income (RMB, CYN)						
< 1000	0.747	0.610-0.915	0.005*	1.671	1.291-2.162	< 0.001*
1000-4999	0.995	0.842-1.176	0.956	1.157	0.968-1.382	0.109
≥ 5000	1			1		
Residence						
Urban	1			1		
Rural	1.636	1.393-1.921	< 0.001*	1.308	1.095-1.563	0.003*
Pre-pregnancy BMI (kg/m^2^)						
< 18.5	1.302	1.081-1.567	0.005	1.237	1.021-1.498	0.030*
18.5-23.99	1			1		
24-27.99	0.599	0.458-0.782	< 0.001*	0.662	0.506-0.867	0.003*
≥ 28	0.460	0.264-0.800	0.006*	0.506	0.283-0.906	0.022*
Parity						
0	1			1		
1-3	2.427	2.075-2.839	< 0.001*	1.071	0.842-1.363	0.576
≥ 4	7.162	3.400-15.089	< 0.001*	2.130	0.920-4.932	0.078

*COR* Crude Odds Ratio, *AOR* Adjusted Odds Ratio, *CI* Confidence interval

*P-value< 0.05 was statistically significant

### The risk of adverse pregnancy outcomes

We enrolled 39,439 women with singleton pregnancies. Table 7 shows the maternal characteristics of the anaemia group and the non-anaemia group. We found that the prevalence of polyhydramnios, preterm birth, low birth weight (< 2500 g), neonatal complications and NICU admission increased in the women with anaemia, while GDM, foetal distress and oligohydramnios increased in non-anaemic women (Table 8).

**Table 7:** Maternal characteristics of the selected population. Data expressed as the means ± SDs or *n* (%)

Variables	Anaemic	Non-anaemic	Total
Pregnant women	6476 (16.4%)	32,963 (83.6%)	39,439 (100%)
Haemoglobin (g/L)	102.16 ± 6.64	125.09 ± 9.64	121.32 ± 12.53
Gestation week	22.65 ± 3.61	23.25 ± 3.36	23.17 ± 3.40
Maternal age (years)	29.21 ± 4.48	29.74 ± 4.18	29.66 ± 4.23
Gravidity	1.99 ± 1.21	1.83 ± 1.13	1.86 ± 1.14
Parity	0.86 ± 0.74	0.59 ± 0.67	0.63 ± 0.69
Pre-pregnancy BMI (kg/m^2^)	20.48 ± 4.51	21.14 ± 4.33	21.04 ± 4.36
Pre-pregnancy height (cm)	160.39 ± 4.92	161.48 ± 4.92	161.30 ± 4.93
Pre-pregnancy weight (kg)	53.96 ± 8.30	55.93 ± 8.89	55.61 ± 8.83

**Table 8:** Adverse pregnancy outcomes in anaemic and non-anaemic women. [*N* (%)]

Variables	Anaemic	Non-anaemic	χ^2^	*P*
Caesarean section	2986(46.1%)	15,031(45.6%)	0.565	0.452
GDM	1031(15.9%)	6575(19.9%)	56.368	< 0.001*
Hypertensive disorder	165 (2.5%)	863 (2.6%)	0.105	0.746
PROM	1319(20.4%)	7021 (21.3%)	2.820	0.093
Foetal distress	611(9.4%)	4119(12.5%)	48.049	< 0.001*
Placenta abruption	28 (0.40)	147(0.4%)	0.23	0.880
Polyhydramnios	125 (1.95)	386 (1.2%)	24.394	< 0.001*
Oligohydramnios	168 (2.6%)	1202 (3.6%)	17.876	< 0.001*
Preterm labour	529 (8.2%)	1600 (4.9%)	116.45	< 0.001*
Low birth weight (< 2500 g)	319 (4.9%)	1108(3.4%)	37.991	< 0.001*
Neonatal complications	687 (10.6%)	2501 (7.6%)	66.489	< 0.001*
NICU admission	631 (9.7%)	2000 (6.1%)	117.492	< 0.001*

^*^P < 0.05 is statistically significant

## Discussion

Anaemia is one of the most common complications during pregnancy and could cause adverse pregnancy outcomes. It is a public health problem not only in developing but also in industrialized countries. In the present study, the overall prevalence of anaemia is 23.5%. According to the WHO classification of the public health importance of anaemia [3], it was a moderate public health problem among the pregnant women in our study. However, compared to the prevalence of anaemia in developed countries [5], it remains a severe health problem in China.

To our knowledge, this study is the first to compare the prevalence of anaemia in three big cities, which may partially represent the Western, Northern and Southern China. We found that the prevalence of anaemia in Guangzhou (38.8%) and Chengdu (23.9%) was significantly higher than the total, while it was lower in Beijing (19.3%). This prevalence was comparable to a study conducted in rural Western China (45.7%) [17]. The results may have been due to the different levels of local economic development, lifestyle, and diet, and may also be related to the altitude of the area.

Our study showed that the prevalence of anaemia increased with the progress of pregnancy. We found that the anaemia prevalence gradually increased from early pregnancy (2.7%) to middle pregnancy (14.7%), then became the highest in late pregnancy (16.6%). The change in the haemoglobin level during the second trimester may be related to physiological changes during pregnancy, which is due to plasma dilution. In the third trimester, physiologically, the increased plasma volume velocity slows down and women may undergo routine antenatal care and iron supplementation, which will elevate the Hb level [18]. However, we found an increased prevalence in the third trimester, which may have been due to inadequate iron supplementation.

Considering the degree of anaemia, Desalegn et al. [19] reported that of 66 anaemic pregnant women, 40.92% had mild, 54.54% had moderate, and 4.54% had severe anaemia. Another study showed that among 224 pregnant women, 37% women had anaemia (26% mild and 11% moderate). Our results showed findings similar to those studies.

The results of our study showed that pregnant women with lower family per capita income were more anaemic than the higher one. The higher prevalence of anaemia was also found among pregnant women from rural areas. The results of a study in Pakistan showed that patients with low income comprised a higher portion of patients with anaemia compared to those with a high income [20]. This is likely related to the lack of information about adequate nutrition during pregnancy, economic factors and the inaccessibility of health care centres. Interestingly, our study also indicated that pre-pregnancy BMI < 18.5 kg/m^2^ was a predictor of anaemia, which may be due to the inadequate nutrition during pregnancy. Previous studies have shown an association of anaemia with low education status [5, 21] and multiparity [22]. However, we did not find this association in our study. This might have been due to variations in the methods and study subjects involved. These predictors of anaemia (including age, income, area, pre-pregnancy BMI) may provide clinical guidance. Women with these risk factors should appropriately increase their nutrition during pregnancy, and pregnant women diagnosed with anaemia should take iron supplements.

It has been suggested that anaemia in pregnancy is associated with an increased risk of adverse pregnancy outcomes, such as preterm birth, hypertensive disorders, and low birth weight [13, 23, 24]. Preterm labour and low birth weight have been reported to be sub-optimal pregnancy outcomes of anaemia in previous studies [8, 25, 26]. These results were in accordance with our findings. We also found that an increase of NICU admission in anaemic women. This may be due to the higher prevalence of preterm birth and low birth weight in anaemic women than non-anaemic women.

The association between GDM and anaemia has not been well reported. In our study, we observed that anaemia reduced the prevalence of GDM. Lao et al. [27] reported that the prevalence of GDM is reduced in iron deficiency anaemia. These results indicate that haemoglobin level is positively associated with the prevalence of GDM. Our study also first reported that anaemia is associated with polyhydramnios, which may occur in parallel with the GDM outcome.

Although the sample size and the study sites included 21 centres in China, there may be bias in our results, as the data were collected in a retrospective manner. Studies reported that the inter pregnancy interval [22] and history of parasitic infection [7, 28] were associated with the prevalence of anemia, which could not be estimated in this study due to the lake of the factors. Recent studies noted that both low and high iron intake was associated with mortality among women [29] and elevated iron level may increase the risk of GDM [30]. However, the present study did not record the iron supplementation of the participants, which may have impacted the results of adverse pregnancy outcomes of anaemia.

## Conclusion

This study showed that anaemia in pregnancy continues to be a health problem in China, and economic factors may contribute to the situation. Therefore, we should vigorously promote early prenatal care for these at-risk pregnant women. This would allow for iron and folic acid supplementation during pregnancy, which would potentially reduce the prevalence of anaemia.

## Additional file

Additional file 1: Questionnaire. (DOCX 104 kb)

## Abbreviations

75 g OGTT: 75 g oral glucose tolerance test
AOR: Adjusted odds ratio
BMI: Body mass index
CI: Confidence interval
CNY: China Yuan
COR: Crude odds ratio
GDM: Gestational diabetes mellitus
GPS study: Gestational diabetes mellitus Prevalence Survey study
Hb: Haemoglobin
HELLP syndrome: Haemolysis, elevated liver enzymes and low platelet syndrome
LMP: Last menstrual period
NICU: Neonatal Intensive Care Unit
OR: Odds ratio
PGDM: Pre-pregnancy diabetes mellitus
PROM: Premature rupture of membranes
SD: Standard deviation
