# Supplementary material for: Prevalence, risk factors and associated adverse pregnancy outcomes of anaemia in Chinese pregnant women: a multicentre retrospective study
Source: BMC Pregnancy Childbirth. 2018 Apr 23;18:111. doi: 10.1186/s12884-018-1739-8 (PMC5914057; doi:10.1186/s12884-018-1739-8)
Supplement: Supplementary file 1 — Questionnaire. (DOCX 104 kb) [file 12884_2018_1739_MOESM1_ESM.docx]

**A Baseline information**

A1 Hospital Name Number

A2 Age year

A3 Education status □

1 College and above 2 Senior or junior high school 3 Primary school or illiteracy

A4 Family monthly income per capita □ 1 <¥1000 2 ¥1000-4999 3 ≥¥5000

A5 Resident area □ 1 Urban 2 Rural

A6 Height cm Self birth weight g Pre-gestational weight kg

**B History**

B1 Pre-pregnancy DM □ 0 NO 1 YES

B2 Hypertension □ 0 NO 1 YES

B3 Other diseases

B3 Other diseases

**C Pregnancy information**

C1 Gravidity Parity

C2 LMP □□/□□/□□ (yy/mm/dd) Adjusted EDC □□/□□/□□ (yy/mm/dd)

C3 Pregnancy history (Please fill “0” for NO or “1” for YES in □)

Macrosomia □ Abortion□ Preterm labor □

C4 Other disease

**D Weight growth during pregnancy**

D1 First-time antenatal care kg ; □□/□□/□□ (yy/mm/dd)

D2 OGTT test kg ; □□/□□/□□ (yy/mm/dd)

D3 Last-time before delivery kg; □□/□□/□□ (yy/mm/dd)

**E Hemoglobin level**

E1 1^st^ trimester g/L; □□/□□/□□ (yy/mm/dd)

E2 2^nd^ trimester g/L; □□/□□/□□ (yy/mm/dd)

E3 3^rd^ trimester g/L; □□/□□/□□ (yy/mm/dd)

**F GDM information**

F1 Diagnosis of diabetes in pregnancy □ 0 NO 1 GDM 2 DM

F2 75g OGTT test (mmol/L)

0h 1h 2h ; □□/□□/□□ (yy/mm/dd)

**G** **Delivery information**

G1 Delivery date □□/□□/□□ (yy/mm/dd)

G2 Delivery way □ 0 Vaginal delivery 1 Cesarean section

G3 Pregnancy outcome (Please fill “0” for NO or “1” for YES in □)

P1 Hypertension disease in pregnancy □

P11 Hypertension in pregnancy □

P12 Mild preeclampsia □

P13 Severe preeclampsia □

P14 Eclampsia □

P15 HELLP syndrome □

P2 Premature rupture of membranes(PROM) □

P3 Placenta abruption □

P4 Polyhydramnios □

P5 Oligohydramnios □

P6 Foetal distress □

P7 Other complications

H0 Foetal number □ 0 Singleton 1 Twin-pregnancy 2 Multiple-pregnancy

Neonatal 1 Neonatal 2

H1 Neonatal birth weight g HH1 Neonatal birth weight g

H2 Neonatal gender □ 0 Female 1 Male HH2 Neonatal gender □ 0 Female 1 Male

H3 Neonatal outcomes □ HH3 Neonatal outcomes □

1 Normal 2 Complications, 1 Normal 2 Complications,

3 Malformations， 4 Death 3 Malformations, 4 Death

H4 NICU admission □ 0 NO 1 YES HH4 NICU admission □ 0 NO 1 YES

**H Neonatal information**
